# Effect of grain inclusion rates in diets provided to early-weaned calves and steroidal implants utilization on growth performance and carcass characteristics of beef steers

**DOI:** 10.1093/tas/txad068

**Published:** 2023-06-27

**Authors:** Alejandro M Pittaluga, Justin Kieffer, Alejandro E Relling

**Affiliations:** Department of Animal Science, The Ohio State University, Columbus, OH 43210. USA; Department of Animal Science, The Ohio State University, Columbus, OH 43210. USA; Department of Animal Sciences/Interdisciplinary PhD Program in Nutrition, The Ohio State University, Wooster, OH 44692, USA

**Keywords:** beef, corn, energy, feedlot, management, quality

## Abstract

One hundred and twenty-one Angus × SimAngus-crossbred steers (body weight (**BW**) = 159 ± 22 kg) were used to evaluate the effects of different grain inclusion (**GI**) rates in diets provided to early-weaned calves and steroidal implants (**SI**) utilization on growth performance and carcass characteristics, particularly intramuscular fat deposition, of beef steers. The experiment was conducted as a randomized complete block design with a 2 × 2 factorial arrangement of treatments, consisting of two GI rates (35% vs. 58%, dry matter (**DM**) basis), each one associated or not to steroidal implant utilization (no implants vs. 80 mg trenbolone acetate (**TA**) + 16 mg estradiol followed by 120 mg TA + 24 mg of estradiol). After being early-weaned (124 ± 14 d of age), steers were offered an average of 4.5 kg/d (DM basis) of a concentrate-based diet with a greater or lesser GI rate for 60 d. After being fed a concentrate-based diet with different GI rates for 60 d, steers were fed a common backgrounding diet for 56 d and subsequently fed a common high-grain diet until harvested at a constant final BW (620 kg). Steers were not implanted until the beginning of the backgrounding phase and then re-implanted when initiating the finishing phase. Data were analyzed using PROC MIXED in SAS. There were no GI × SI interactions (*P* ≥ 0.62) for any of the growth performance parameters throughout the experimental period. Implanted steers tended to have a greater average daily gain (*P* = 0.10) during the finishing phase than nonimplanted steers. For the 12th rib fat thickness and yield grade (**YG**), a GI × SI interaction (*P* = 0.03) and a tendency for a GI × SI interaction (*P* = 0.10) was detected, respectively. Nonimplanted steers fed diets with greater GI rates presented the greatest 12th rib fat thickness and tended to have the greatest YG among treatments. No other interactions (*P* ≥ 0.33) were observed for the hot carcass weight, *Longissimus* muscle (**LM**) area, quality grade, marbling score, and kidney-pelvic-heart fat content. Steers fed diets with lesser GI rates tended to have a greater LM area than steers fed diets with greater GI rates (*P* = 0.10). Results from this experiment indicate that varying GI rates in diets provided to early-weaned calves and subsequent implantation with steroidal hormones did not affect marbling deposition.

## Introduction

In beef cattle, intramuscular adipogenesis has been suggested to take place between late fetal stages and 250 d after birth (Wang et al., 2009; Du et al., 2015). Since adipogenesis in the other fat depots concludes at early postnatal stages, before 250 d, an opportunity window arises to enhance intramuscular fat (**IMF**) or marbling deposition without an overall increase in fatness prior to reaching conventional weaning ages (Du et al., 2013). To effectively capitalize this opportunity window into more marbling, identifying the best nutritional management to stimulate intramuscular adipogenesis is necessary. Based on evidence suggesting that glucose is preferentially used as a carbon donor for fatty acid synthesis in the IMF tissue instead of ruminal acetate (Smith and Crouse, 1984; Rhoades et al., 2007), starch-based diets that increase the molar proportions of ruminal propionate might increase the IMF deposition during this stage. Ruminal propionate has been defined as the main gluconeogenic precursor in ruminants (McAtee and Trenkle, 1971). However, recent reports showing that the IMF tissue uses mainly ruminal acetate as a substrate for de novo lipogenesis (Nayananjalie et al., 2015a; Smith et al., 2018) blurs the role of glucose in marbling deposition.

Earlier studies reported no benefit of early grain feeding to beef calves in marbling deposition (Myers et al., 1999; Fluharty et al., 2000; Schoonmaker et al., 2003). However, recent findings showed that early-weaned calves fed diets with 35% to 41% of cracked corn had a greater marbling score (**MS**) compared to their normal weaned contemporaries harvested at a common 12th rib fat thickness (Nayananjalie et al., 2015b). Early grain feeding to beef calves induced a precocious activation of the peroxisome proliferator-activated receptor gamma and its target genes in the *Longissimus* muscle (**LM**) leading to a greater IMF deposition than normal weaned calves (Moisá et al., 2014). Furthermore, the activation of key adipogenic activators was sustained throughout the animal’s life, likely due to the metabolic imprinting effects of early grain feeding (Moisá et al., 2014; Scheffler et al., 2014). Nevertheless, compared to normal weaned calves conventionally managed, it is not clear whether the greater MS of early-weaned calves was due to the inclusion of starch as the main energy source or to a greater daily energy intake. In this context, utilizing steroidal implant (**SI**) that prioritizes muscle growth rather than adipogenesis might suppress further upregulation of adipogenic activators and mask potential improvements in MS. In addition, implanting beef cattle with SI has been suggested to decrease the MS due to intramuscular fat dilution via increased LM area (Smith and Johnson, 2020).

We hypothesized that 1) feeding diets with a greater grain inclusion (**GI**) rate (58% of the dry matter [**DM**] basis) to early-weaned calves will improve the MS compared to feeding diets with a lesser GI rate (35% of the DM basis) to early-weaned calves, and 2) early-weaned steers fed diets with a greater GI rate (58% of the DM basis) that are not implanted with SI after initiating the backgrounding phase will present the greatest MS among treatments. Our objectives were to investigate the effects of different GI rates in diets provided to early-weaned calves and SI utilization after initiating the backgrounding phase on growth performance and carcass characteristics of beef steers.

## Materials and Methods

All experimental procedures were approved by the Institutional Animal Care and Use Committee of The Ohio State University (2021A00000035) and followed the guidelines recommended in the Guide for the Care and Use of Agricultural Animals in Research and Teaching (FASS, 2010).

### Animals, Experimental Design, and Treatments

Angus × SimAngus-crossbred steers (*n* = 120; initial body weight (**BW**) = 159 ± 22 kg) were used in a randomized complete block design with a 2 × 2 factorial arrangement of treatments. Steers were born from February to April 2021 and suckled their dams while grazing endophyte-infected tall fescue pastures (*Festuca arundinacea* L.) until the time of early weaning (124 ± 14 d of age) when they were blocked by age (early-born and late-born), stratified by BW, and randomly assigned to one of four treatments (*n* = 2 pens/treatment; 15 steers/pen). Treatments consisted of early-weaned steers fed either a concentrate-based diet (Table 1) with 58% (DM basis) of whole shelled corn (**WSC**) with (**Hi/Imp**) or without (**Hi/NI**) SI, or a concentrate-based diet with 35% (DM basis) of WSC with (**Lo/Imp**) or without (**Lo/NI**) SI. Diets provided to early-weaned calves were formulated to be isoenergetic by increasing the proportion of distillers grains in partial replacement of alfalfa hay and corn grain in the diet with a lesser GI rate. Steers were vaccinated with Ultrachoice 7 (Pfizer Animal Health, Parsippany-Troy Hills, NJ) and with Autogenous Bacterin Maxi/Guard (Addison Biological Laboratory Inc., Fayette, MO) at weaning. Steers were housed in pens (7.3 × 37.2 m) that included an area covered by a metal roof (7.3 × 8.5 m) and an outside loafing area (7.3 × 28.6 m). The flooring material under the covered space was comprised of crushed, compacted limestone (screenings), and the outside loafing area was concrete.

**Table 1.** Ingredients and analyzed nutrient content of experimental diets

Item	Lesser GI	Greater GI	Backgrounding	Finishing
Ingredient, % of DM				
Whole shelled corn	35.040	58.000	—	—
Dry rolled corn	—	—	29.960	70.160
DDGS^a^	38.000	14.000	13.000	18.000
Soy hulls	15.500	4.000	30.000	—
Alfalfa hay	8.000	14.000	—	—
Grass hay	—	—	20.000	8.000
Urea	—	0.540	0.539	0.369
Soybean meal	—	6.423	3.041	—
Limestone	1.659	1.301	1.659	1.661
Salt	0.461	0.361	0.461	0.462
Vitamin A, 30,000 IU/g	0.007	0.005	0.007	0.007
Vitamin D, 3,000 IU/g	0.007	0.005	0.007	0.007
Vitamin E, 44 IU/g	0.021	0.016	0.021	0.021
Calcium sulfate	0.645	0.506	0.645	0.646
Selenium	0.035	0.027	0.035	0.035
Rumensin 90^b^	0.016	0.012	0.016	0.020
Potassium chloride	0.277	0.217	0.277	0.277
Copper sulfate	0.006	0.004	0.006	0.006
Zinc sulfate	0.018	0.015	0.017	0.018
Manganese sulfate	0.009	0.007	0.008	0.010
Cobalt carbonate	0.000	0.000	0.001	0.001
AV blend^c^	0.300	0.560	0.300	0.300
Analyzed composition, % of DM				
CP^d^	18.21	16.54	15.39	13.02
NDF^e^	29.07	19.39	43.76	24.96
EE^f^	2.45	3.16	2.40	2.56
Starch	26.79	42.69	22.28	51.56
Ash	6.95	5.23	6.71	4.99
NEm^g^, Mcal/kg	1.89	1.95	1.65	1.95
NEg^h^, Mcal/kg	1.25	1.30	1.04	1.30

^a^Distillers dry grain with solubles.

^b^Contained 200 g Monensin/kg.

^c^Animal-Vegetable fat blend.

^d^Crude protein.

^e^Neutral detergent fiber.

^f^Ether extract.

^g^NE_m_ = Estimated net energy for maintenance.

^h^NE_g_ = Estimated net energy for gain.

After a 24-d adaptation period, where alfalfa hay was replaced with WSC in a step-wise fashion, steer calves were offered an average of 4.5 kg/d (DM basis) of the concentrate-based diet with a greater or lesser GI rate for 60 d until steers reached conventional weaning ages (208 ± 14 d). After being fed a concentrate-based diet with different GI rates for 60 d, steers were fed a common backgrounding diet for 56 d. Upon finalization of the backgrounding phase, steers were fed a dry rolled corn-based finishing diet for ad libitum intake until reaching harvesting target (620 kg of final BW). Steers were not implanted until the beginning of the backgrounding phase, when they were implanted with Component TE-IS (80 mg trenbolone acetate, 16 mg estradiol; Elanco Animal Health, Indianapolis, IN), and then re-implanted when initiating the finishing phase with Component TE-S (120 mg of trenbolone acetate, 24 mg of estradiol; Elanco Animal Health). Individual feed intake was only collected during the finishing phase through the GrowSafe (GrowSafe, GrowSafe Systems Ltd, Airdrie, AB, Canada) feeding systems bunks (0.91 m × 0.53 m × 0.38 m). Steers were adapted to the GrowSafe bunks over a 7-d period prior to transitioning from the backgrounding to the finishing diet. All experimental diets were fortified to provide vitamins and minerals to meet or exceed nutrient requirements (NASEM, 2016). A total of three steers (Hi/Imp = 1, Hi/NI = 1, Lo/NI = 1) were removed from the experiment as a result reasons irrelative to the experiment.

### Data Collection and Laboratory Analysis

Steers were weighed on two consecutive days at the beginning and end of the experiment, and then every 28-d throughout the experiment before the morning feeding without withdrawal from feed and water. Upon reaching the harvesting target BW, steers were transported for 2 h in a commercial truck to a commercial abattoir (E.R. Boliantz Co., Ashland, OH) for harvest. Carcass data were provided by a USDA grader. Carcasses were chilled for 48 h at −4 °C and posteriorly ribbed between the 12th and 13th ribs to determine 12th rib fat thickness, kidney-pelvic-heart (**KPH**) fat (subjectively estimated), MS, LM area, and yield grade (**YG**). The USDA assigned YG (calculated to the nearest 0.1) was calculated using the USDA regression equation (USDA, 2016), where YG = 2.5 + (2.5 × 12th rib fat thickness) + (0.2 × % KPH fat) + (0.0038 × hot carcass weight (**HCW**)) − (0.32 × LM area).

Feed ingredient samples were collected biweekly throughout the experiment and frozen at −20 °C. At the end of the experiment, samples were thawed, and equal portions of each ingredient were composited and shipped for nutrient composition analysis (Rock River Laboratory Inc., Agricultural Analysis; Wooster, Ohio, OH). Composite samples were dried and ground through a Wiley mill (1 mm screen, Arthur H. Thomas, Philadelphia, PA). Ingredients were analyzed for dry matter by oven-drying (24 h at 105 °C ). Neutral detergent fiber (**NDF**) and acid detergent fiber (**ADF**) were analyzed using an ANKOM 200 fiber analyzer (ANKOM Technology Corporation) according to the ANKOM Technology methods 5 and 6, respectively. Ether extract (**EE**) was determined using the ANKOM filter bag analysis system according to procedure AM 5 − 04 (AOAC, 2002). Total nitrogen was analyzed using a LECO TruMac N Nitrogen Determinator (LECO Corporation, St. Joseph, MI) according to AOAC method (AOAC, 1997; #990.03). The crude protein (**CP**) content was calculated as N × 6.25. Starch was analyzed by wet chemistry using the method of Hall (2009), including the use of acetate buffer and correction for free glucose. The ash content was determined by ignition of samples at 600 °C for 2 h using a Thermolyte muffle oven Model F30420C (Thermo Scientific, Waltham, MA) according to the AOAC method (AOAC, 2005; #942.05). The net energy content of the diets was estimated based on their respective analyzed nutrient composition (NASEM, 2016).

### Statistical Analysis

All statistical analyses were conducted using PROC MIXED in SAS 9.4 (SAS Inst. Inc., Cary, NC). Growth performance and carcass characteristics were analyzed as a randomized complete block design with a 2 × 2 factorial arrangement of treatments with pen as the experimental unit. The model was fitted with individual animal data and included the GI rate, SI utilization, and their interaction as fixed effects, as well as the random effects of the block and pen within the block. The initial BW was used as a covariate to account for BW differences at the beginning of the finishing phase and the final BW. The LS-means were separated using the PDIFF and SLICE option of SAS. Significance was declared at *P* ≤ 0.05 and tendencies were discussed at 0.05 < *P* ≤ 0.10.

## Results and Discussion

### Growth Performance

No effect (*P* = 0.88) of the GI rate in diets provided to early-weaned calves was observed in the average daily gain (**ADG**) of steers prior to the beginning of the backgrounding phase. The ADG (± SEM) was 0.75 and 0.74 kg/d for steers fed diets with a greater and lesser GI rate, respectively (± 0.02). No effect (*P* = 0.73; Table 2) of the GI rates in diets provided to early-weaned calves was observed in the BW of steers at the beginning of the backgrounding phase. The BW (± SEM) was 224 and 223 kg for steers fed diets with a greater and lesser GI rate, respectively (± 1.6). There were no GI × SI interactions for any of the growth performance parameters throughout the experimental period (*P* ≥ 0.62). Implanted steers tended to have a greater ADG (*P* = 0.10) during the finishing phase than non-implanted steers. No other treatment effects were detected (*P* ≥ 0.16) for the growth performance parameters.

**Table 2.** Effect of different grain inclusion (GI) rates in diets provided to early-weaned calves and steroidal implants (SI) utilization on the growth performance of beef steers

Item	Treatment^a^				SEM^b^	*P*-values		
	Hi/Imp	Hi/NI	Lo/Imp	Lo/NI		GI	SI	GI*SI
*n*	30	30	31	30				
BW, kg								
Initial BW	157	160	158	163	4.8	0.50	0.36	0.78
Finishing^c^	306	313	310	314	4.3	0.45	0.16	0.66
Final BW	616	619	627	622	10.5	0.33	0.90	0.62
ADG^d^, kg/d								
Backgrounding	1.55	1.56	1.63	1.62	0.05	0.16	0.96	0.83
Finishing	1.61	1.54	1.65	1.59	0.05	0.25	0.10	0.92
Overall	1.37	1.35	1.41	1.38	0.04	0.25	0.54	0.86
DMI^e^, kg/d	10.1	9.9	10.3	10.2	0.25	0.25	0.80	0.84
G:F^f^	0.161	0.155	0.160	0.157	<0.01	0.89	0.17	0.77
DOF to harvest^g^	198	200	197	192	5	0.50	0.62	0.68

Significance was declared at *P* ≤ 0.05; and tendencies were declared at *P* > 0.05 and *P* ≤ 0.10.

^a,b^Within a row, means without a common superscript differ.

^a^Steers fed early weaning diets with greater inclusion rate of grain with (Hi/Imp) or without (Hi/NI) being subsequently implanted, and steers fed early weaning diets with lesser inclusion rate of grain with (Lo/Imp) or without (Lo/NI) being subsequently implanted.

^b^Pooled standard error of treatments means.

^c^Body weight of steers at the beginning of the finishing phase.

^d^Average daily gain.

^e^Dry matter intake.

^f^Gain-to-feed ratio.

^g^Days on feed in the finishing phase required to reach harvesting target.

By design, no treatment differences were expected in the ADG of steer calves before the backgrounding phase as diets were isoenergetic and offered at equal daily amounts. Following implantation, steers treated with SI were expected to have a greater ADG compared to the non-implanted cohort as indicated by the preponderance of available data for cattle treated with growth-enhancing technologies (Smith and Johnson, 2020). During the backgrounding phase, when steers were implanted with a SI of a lower anabolic potency, feeding a more energetically diluted diet with a high NDF content might explain the absence of differences in ADG between implanted and nonimplanted steers. When nutrients, primarily protein and/or energy are limiting, the impact of SI in cattle performance is restricted (Johnson and Beckett, 2014). Subsequently, during the finishing phase, the lower BW of implanted steers observed at the beginning of the backgrounding was mitigated due to their tendency to express a greater ADG. Nevertheless, the magnitude of the improvements in the ADG of implanted cattle (~4.2%) falls outside the 8% to 28% range of improvements usually reported for beef cattle treated with SI (Smith and Johnson, 2020). Moderate increases in dry matter intake (**DMI**) often contribute with improvements in growth performance of cattle when implanted with steroidal hormones (Duckett and Pratt, 2014). In this study, during the finishing phase, decreases in the DMI of steers as a result of feed overconsumption associated with the ad libitum feeding regime might have limited their response to implantation. Restricted feeding programs and feed bunk managements, which are commonly applied in trials that evaluate the effect of SI utilization in cattle productivity, have been proposed as effective strategies to reduce the feed overconsumptions observed with ad libitum regimes (Pritchard and Bruns, 2003). Feed overconsumption frequently leads to digestive upsets and lower daily feed intakes (Galyean, 1999). Despite improvements in the ADG of cattle as a result of SI utilization are regularly accompanied by greater feed efficiencies (Nichols et al., 2002), the absence of differences in the gain-to-feed ratio (**G:F**) between non-implanted and implanted steers observed herein agrees with previous literature (Carvalho et al., 2020).

### Carcass Characteristics

There was a treatment interaction (*P* = 0.03; Table 3) and a tendency for a treatment interaction (*P* = 0.10) for the 12th rib fat thickness and the YG, respectively. Implanting steers with SI decreased the 12th rib fat thickness and tended to decrease the YG among steers fed diets with greater GI rates at weaning but not within steers fed diets with lesser GI rates. No other interactions were detected for the HCW, LM area, quality grade (**QG**), MS, and KPH fat content (*P* ≥ 0.33). Compared to a greater GI rate, a lesser GI rate in the diet provided to early-weaned calves tended to increase the LM area (*P* = 0.10). No other treatment effects were observed for the HCW, QG, MS, and KPH fat content (*P* ≥ 0.18).

**Table 3.** Effect of different grain inclusion (GI) rates in diets provided to early-weaned calves and steroidal implants (SI) utilization on the carcass characteristics of beef steers

Item	Treatment^a^				SEM^b^	*P*-values		
	Hi/Imp	Hi/NI	Lo/Imp	Lo/NI		GI	SI	GI*SI
*n*	30	30	31	30				
HCW^c^, kg	380	384	391	390	6.7	0.18	0.86	0.71
LM area^d^, cm^2^	94.9	92.5	97.2	96.3	2.3	0.10	0.37	0.65
12th rib fat, cm	1.90^b^	2.25^a^	1.96^b^	1.89^b^	0.10	0.09	0.13	0.03
MS^e^	664	681	662	680	23	0.92	0.37	0.96
KPH^f^, %	2.71	2.76	2.73	2.79	0.08	0.72	0.47	0.99
YG^g^	3.4^b^	3.8^a^	3.4^b^	3.4^b^	0.1	0.14	0.06	0.10
QG^h^	6.3	6.5	6.2	6.3	0.2	0.49	0.52	0.76

Significance was declared at *P* ≤ 0.05; and tendencies were declared at *P* > 0.05 and *P* ≤ 0.10.

^a,b^Within a row, means without a common superscript differ.

^a^Steers fed early weaning diets with greater inclusion rate of grain with (Hi/Imp) or without (Hi/NI) being subsequently implanted, and steers fed early weaning diets with lesser inclusion rate of grain with (Lo/Imp) or without (Lo/NI) being subsequently implanted.

^b^Pooled standard error of treatments means.

^c^Hot carcass weight.

^d^ *Longissimus* muscle area.

^e^Marbling score; scale: 400 to 490 = slight, 500 to 590 = small, 600 to 690 = modest, 700 to 790 = moderate, 800 to 890 = slightly abundant.

^f^Kidney-pelvic-heart fat.

^g^Yield grade.

^h^Quality grade: 4 = Select, 5 = Low choice, 6 = Average choice, 7 = High choice.

Our hypothesis that including starch at greater rates during the early weaning phase was going to improve the MS of steers was not confirmed. Results from this experiment disagree with those that reported improvements in MS as a result of feeding starch-based diets to early-weaned calves (Meyer et al., 2005; Moisá et al., 2014; Nayananjalie et al., 2015b). Compared to these last experiments, the lesser ADG of steers observed in the present study during the early weaning phase, due to the limited amounts of feed offered daily, might partially explain such discrepancies. Insufficient energy intakes to support a greater adipogenic activity could have hindered responses of the **IMF** tissue to greater GI rates. Alternatively, differences in the GI rates in the diets provided to early-weaned claves in our study were plausibly not wide enough to elicit adipogenic responses in the IMF tissue. Furthermore, it is also possible that intramuscular adipogenesis relies mainly on the energy intake rather than on the type of energy substrate, thus adding more starch to the diet might not be an effective method to differentially promote fat accretion at the intramuscular depot in vivo. In addition, recent studies showing that the IMF tissue uses more acetate as a substrate for de novo lipogenesis when compared with glucose (Nayananjalie et al., 2015a; Smith et al., 2018) supports the MS results observed herein. To the authors’ knowledge, the negative effect of greater GI rates in diets provided to early-weaned steers in the LM area has not been previously reported. The underlying mechanisms that explain the tendency for a lower LM area of steers fed diets with greater GI at the time of early weaning warrants further research.

Regarding the effect of SI on carcass characteristics, the use of implants usually decreases the 12th rib fat thickness and MS, and increases the LM area over nonimplanted steers harvested at equal final BW (Guiroy et al., 2002; Smith and Johnson, 2020). In the present study, the lack of differences in the 12th rib fat thickness, MS, and LM area is probably a consequence of the slight response in growth performance of steers to implantation.

In conclusion, feeding diets with greater inclusion rates of corn grain to early-weaned calves did not lead to improvements in the marbling score of beef steers. However, more divergent starch contents in diets provided to early-weaned calves managed in a nonrestricted feeding regime might be necessary to elicit a differential response of the intramuscular adipose tissue to the type of energy source. Implanting steers with steroidal hormones of lesser anabolic potency at the beginning of the backgrounding phase and subsequent re-implanting with a more potent combination of steroidal hormones had no effect on marbling deposition.
